# Clinical impact of a new cardiac magnetic resonance imaging program: a single center US experience

**DOI:** 10.1186/1532-429X-14-S1-P42

**Published:** 2012-02-01

**Authors:** Siddique Abbasi, Jaehoon Chung, Ankit A Desai, Afshin Farzaneh-Far

**Affiliations:** 1 Division of Cardiology, University of Illinois at Chicago, Chicago, IL, USA; 2 Division of Cardiology, Duke University, Durham, NC, USA

## Summary

While establishing a Cardiac Magnetic Resonance (CMR) program at an academic institution in the United States, we sought to evaluate indications and direct clinical impact of CMR on patient management and decision-making.

## Background

CMR provides a wealth of unique diagnostic and prognostic information in patients with cardiovascular disease. However, little is known about the direct additive effects of information derived from CMR with respect to clinical decision making. Although there is preliminary data suggesting significant impact of CMR on clinical management, this has been derived entirely from European centers where routine use of CMR is significantly more widespread than in the United States. In the current health care environment in the United States, greater use of a new imaging modality like CMR requires evidence for direct additive impact on clinical management.

## Methods

This was a single-center registry from an academic medical center in the United States. The first 200 patients from a newly established CMR program were enrolled. All procedures were performed using standardized SCMR-recommended protocols on 1.5T and 3T scanners and interpreted by a Level 3 reader. Definitions for “significant clinical impact” of CMR were pre-defined and were collected directly from medical records and/or from patients. Categories of clinical impact included: new diagnosis, medication change, hospital admission/discharge, as well as performance or avoidance of invasive procedures (angiography, revascularization, device therapy or biopsy).

## Results

The most common indications for referral were cardiomyopathy/myocarditis (28%), ischemic heart disease/viability testing (19%), suspected infiltrative disease (11%), and cardiac mass/thrombus (8%). Nearly all patients (96%) had recent prior echocardiograms. In 48% of patients, CMR had a significant clinical impact. This included an entirely new diagnosis in 25% of cases and a change in medication management in 12%. CMR results directly led to angiography in 5% and to performance of percutaneous coronary intervention in 5%. Bypass surgery was performed in 2% and avoided in 5%, directly as a result of CMR findings.

## Conclusions

This experience demonstrates the ability of a new CMR program to significantly impact clinical decision-making at an academic medical center in the United States. In approximately half of all patients referred, CMR led to a meaningful change in clinical management. This additive impact was seen despite widespread use of echocardiography and other imaging tests. This study lends support to the growing use of CMR in the United States.

## Funding

None. No author disclosures.

